# COVID-19 Vaccination Safety and Tolerability in Patients Allegedly at High Risk for Immediate Hypersensitivity Reactions

**DOI:** 10.3390/vaccines10020286

**Published:** 2022-02-14

**Authors:** Toon Ieven, Martijn Vandebotermet, Lisa Nuyttens, David Devolder, Peter Vandenberghe, Dominique Bullens, Rik Schrijvers

**Affiliations:** 1 Department of General Internal Medicine, Division of Allergy and Clinical Immunology, University Hospitals Leuven, 3000 Leuven, Belgium; toon.ieven@uzleuven.be (T.I.); martijn.vandebotermet@uzleuven.be (M.V.); 2 Allergy and Clinical Immunology Research Group, Department of Microbiology, Immunology and Transplantation, Katholieke Universiteit Leuven, 3000 Leuven, Belgium; lisa.nuyttens@kuleuven.be (L.N.); dominique.bullens@uzleuven.be (D.B.); 3 Department of Pediatrics, University Hospitals Leuven, 3000 Leuven, Belgium; 4 Pharmacy Department, University Hospitals Leuven, 3000 Leuven, Belgium; david.devolder@uzleuven.be; 5 Department of Hematology, University Hospitals Leuven, 3000 Leuven, Belgium; peter.vandenberghe@uzleuven.be; 6 Laboratory for Genetics of Malignant Disorders, KU Leuven Department of Human Genetics, Katholieke Universiteit Leuven, 3000 Leuven, Belgium

**Keywords:** allergy, anaphylaxis, COVID-19, hereditary angioedema, hypersensitivity, mastocytosis, SARS-CoV-2, vaccination

## Abstract

The reported incidence of immediate hypersensitivity reactions (IHR) including anaphylaxis after COVID-19 vaccination is 10-fold higher than for other vaccines. Several patient groups are theorized to be at particular risk. Since specific vaccination guidelines for these patients are based on expert opinion, we performed a retrospective monocentric analysis of the tolerability of adenoviral vector and mRNA-based COVID-19 vaccines in a cohort of patients allegedly at high risk of IHR. Reactions were assessed immediately on-site by allergists during a monitored vaccination protocol and after 3–7 days through telephone interviews. The cohort included 196 patients (aged 12–84 years) with primary mast cell disease (pMCD, 50.5%), idiopathic anaphylaxis (IA, 19.9%), hereditary angioedema (HAE, 5.1%) or miscellaneous indications (24.5%). Twenty-five immediate reactions were observed in 221 vaccine doses (11.3%). Most occurred in IA or miscellaneous patients. None fulfilled anaphylaxis criteria and most were mild and self-limiting. Reaction occurrence was significantly associated with female sex. In total, 13.5% of pMCD patients reported mast cell activation-like symptoms within 72 h post-vaccination. All pediatric pMCD patients (*n* = 9, 12–18 years) tolerated both mRNA-based vaccine doses. In summary, adenoviral vector and mRNA-based COVID-19 vaccines were safe and well-tolerated in patients with pMCD, HAE, and IA. No anaphylaxis was observed. The mild and subjective nature of most reactions suggests a nocebo effect associated with vaccination in a medicalized setting. Patients with pMCD could experience mild flare-ups of mast cell activation-like symptoms, supporting antihistamine premedication.

## 1. Introduction

The reported incidence of severe immediate hypersensitivity reactions (IHR) including anaphylaxis after COVID-19 vaccination is unexpectedly high. For the new mRNA-based COVID-19 vaccines in particular, this incidence is estimated at 5–10 per million doses or 10 times higher than for other vaccines [1,2]. These data are based on optional reporting by patients and physicians through passive reporting systems such as the U.S. Vaccine Adverse-Events Reporting System (VAERS) and must be interpreted with care [3].

The mechanism for COVID-19 vaccine-related IHR remains unclear. IgE sensitization to structurally related excipients such as polyethylene glycol (PEG) and polysorbate 80 (PS80) have been implicated in a subset of vaccine reactors [4,5]. In addition, non-IgE-mediated mechanisms such as direct contact system activation, complement activation-related pseudo allergy (CARPA), and direct mast cell activation by mRNA vaccine lipid nanoparticles, have been put forward. Based on these hypothetical mechanisms, it has been theorized that certain conditions predisposing for mast cell activation, anaphylaxis, and/or angioedema may increase the risk of vaccine-related IHR [6].

Advisory agencies have issued specific warnings and guidelines concerning vaccination of patients judged to be at particular risk for IHR. The breadth and scale of these warnings as well as the definition of this risk vary considerably. In Belgium, the Superior Health Council stated in January 2021 that administration of the first COVID-19 vaccine dose in patients at high risk for IHR should take place in-hospital under allergist supervision [7]. This population was defined as all patients with a primary mast cell disorder (pMCD), history of idiopathic anaphylaxis (IA), or hereditary angioedema (HAE). In addition, patients with a confirmed history of IHR to COVID-19 vaccine excipients or possible IHR after vaccination with vaccines containing similar excipients were advised to consult an allergist for guidance. Since data on the true incidence of vaccine-related IHR in these populations is lacking, the Belgian guidelines were expert-based and deviated from guidelines issued in other countries [8,9,10].

Despite patients with pMCD being considered at particular risk for anaphylaxis, data on vaccine tolerability and safety in this group are limited. In a recent position paper, the American Initiative on Mast Cell Diseases (AIM) and European Competence Network on Mastocytosis (ECNM) advised prolonged monitoring and antihistamine premedication of pMCD patients receiving a COVID-19 vaccine, while acknowledging a lack of evidence [11].

In this study we analyzed the allergist-observed safety and tolerability of COVID-19 vaccines in patients deemed at higher risk for COVID-19 vaccine-induced IHR, vaccinated in-hospital under medical supervision, to guide further vaccination strategies and determine the need for risk stratification, vaccine selection, premedication, and monitoring.

## 2. Materials and Methods

We performed a single-center retrospective cohort study of patients vaccinated in hospital. The study protocol was approved by the Ethics Committee Research of University Hospitals Leuven with a waiver of informed consent (S65711).

### 2.1. Patient Selection

All patients in follow-up or referred to our tertiary referral center, identified to be at high risk for IHR upon COVID-19 vaccination according to the criteria established by the Belgian Superior Health Council, were invited for vaccination under medical supervision in the hospital vaccination unit. Inclusion occurred until 18 July 2021 for adults and 1 October 2021 for children. High-risk groups were defined as follows:

1. Primary mast cell disorders (pMCD), including cutaneous (CM) or systemic mastocytosis (SM) diagnosed according to World Health Organization (WHO) criteria and primary mast cell activation syndrome (pMCAS) defined according to the criteria proposed by Valent et al. [12,13]. Patients with proven cutaneous involvement who had not undergone bone marrow analysis to rule out systemic involvement at time of inclusion, including the majority of pediatric pMCD cases, were labeled with the intermediate diagnostic label of mastocytosis in the skin (MIS), defined according to expert consensus [13].
2. Idiopathic anaphylaxis (IA), defined as recurrent anaphylaxis without identifiable triggers despite extensive allergy workup or evidence of underlying pMCD.
3. Hereditary angioedema (HAE), defined according to the World Allergy Organization (WAO)/European Academy of Allergy and Clinical Immunology (EAACI) guidelines [14].
4. A miscellaneous group, consisting of (a) patients with a history of immediate reactions to other vaccines; (b) patients with (non)-anaphylactic reactions to the first dose of any COVID-19 vaccine; (c) patients with a proven PEG allergy of whom 10/11 were reported in a previous study [15]; and (d) others, amongst which patients with a history of isolated anaphylaxis-like or poorly circumscribed severe reactions to drug(s) or unclear trigger(s) not fulfilling the criteria for IA.

### 2.2. Vaccine Selection

The mRNA-based BNT162b2 vaccine (Comirnaty^®^, Pfizer-BioNTech, New York, NY, USA) and adenoviral vector Ad26.COV2-S vaccine (COVID-19 Vaccine Janssen^®^, Johnson & Johnson, New Brunswick, NJ, USA) were used for in-hospital vaccination depending on availability and patient profile.

For practical and safety reasons, all eligible patients were preferentially vaccinated with the single-dose Ad26.COV2-S vaccine.

In patients with a possible or confirmed excipient allergy, an additional workup was performed prior to vaccination as previously described to decide on the selection of either a PEG-containing (BNT162b2) or PS80-containing vaccine (Ad26.COV2-S) [15].

Due to reports of thrombo-embolic complications related to adenoviral vector vaccines (Ad26.COV2-S and ChAdOx1; Vaxzevria^®^, Oxford-AstraZeneca, Cambridge, UK), in April 2021, the Belgian Federal Health Service restricted these vaccines to patients aged 41 years or older. Therefore, most patients under 41, including all pediatric patients, received BNT162b2. In selected cases and upon indication, Ad26.COV2-S could still be administered in patients under 41 years, provided informed consent was obtained.

### 2.3. Vaccination Procedure

Vaccination was performed from 25 May until 1 October 2021 by trained nursing staff under allergist supervision in the hospital vaccination unit, close to the emergency department (ED). A standard operating procedure outlining the vaccination protocol was generated to limit variability and was approved by all relevant stakeholders (see online supplement). Resuscitation equipment and rescue medication was available on-site. Patients remained in-hospital for an obligatory 30 min observation period before discharge based on the Aldrete scoring system [16]. If deemed necessary, possibility for extended monitoring up to 2 h on-site was provided. Patients requiring monitoring for longer periods were transferred to the ED. The clinical course of all observed reactions and administered rescue medication were registered in a standardized manner.

In accordance with society guidelines, pMCD patients were advised to start H1-antihistamines or temporarily increase their maintenance dose from 3 days before up to 2 days after vaccination. All patients were advised to bring their regular rescue medication (adrenalin autoinjector or HAE emergency medication).

### 2.4. Tryptase Measurement

Serum tryptase is a biomarker for mast cell burden and degranulation. A transient increase in tryptase in the first hours after a potential IHR is indicative of an underlying mast cell-mediated mechanism such as IgE-mediated anaphylaxis [6]. In order to assess potential mast cell involvement and guide subsequent management and diagnostic evaluation, acute tryptase levels were obtained 1–2 h after reaction onset in patients with reactions deemed to be severe. Due to low intra-patient variability, baseline tryptase levels for comparison were obtained from previous patient records or at least 24 h after the reaction if not available in the records. Tryptase assays were performed in our hospital’s clinical laboratory, with a normal range of 1–11 µg/L and reporting range of 1–200 µg/L as indicated by the manufacturer (ImmunoCAP, Phadia, Uppsala, Sweden). Significant tryptase elevation was defined according to a validated formula as an increase of 20% + 2 µg/L compared to baseline [17].

### 2.5. Data Collection

Patient data (age, sex, underlying diagnosis, history of anaphylaxis and mast cell activation symptoms (MCAS), baseline serum tryptase, c-KIT D816V mutation status) were collected for all patients.

Occurrence of immediate reactions (IR) was assessed on-site by the supervising allergist (M.V., D.B., R.S.) and clinical fellow (T.I., L.N.). Clinical signs, symptoms, and vital parameters were recorded in the electronic patient record. An IR was defined broadly as any event occurring within 30 min after vaccination observed by the supervising clinician during the in-hospital observation period. IR were classified as severe (sIR) when they required extended observation for >2 h based on the supervising allergist’s judgement. Anaphylaxis was defined according to the Brighton collaboration and WAO criteria [18,19].

A standardized follow-up telephone interview was conducted by a physician (T.I.) or clinical trial assistant within 3–7 days after the first in-hospital vaccine dose. Compliance with premedication, occurrence of late reactions (LR) including all patient-reported symptoms, and any self-administered rescue medication in the period following hospital discharge were recorded.

### 2.6. Statistical Analysis

Statistics were performed using IBM SPSS version 28. Frequencies were calculated for nominal variables; medians and ranges for continuous variables. Relationships between IR or LR occurrence and other clinical variables were calculated for nominal variables using Fisher’s exact whenever possible or Pearson’s Chi-square alternatively, and for nominal and continuous variables with the Mann–Whitney U test. Statistical significance was set at *p* < 0.05.

## 3. Results

### 3.1. Study Population Overview and Vaccine Distribution

In total, 196 allegedly high-risk patients were vaccinated in-hospital during the study period, including 99 patients with pMCD, 39 with IA, 10 with HAE, and 48 with miscellaneous indications (Table 1 and Supplementary Material Table S1). The majority of high-risk patients were women (65.3%) and the median age was 51.5 years (range 12–84). Median baseline tryptase across the entire cohort was 8.5 µg/L (*n* = 163; range 1.5–200). A prior history of anaphylaxis was noted in 53.1% of patients. The most frequent anaphylaxis triggers noted in this group were hymenoptera venom in 25.9% and PEG compounds of varying molecular weight in 10.6% of patients. Over half of patients with a history of anaphylaxis (54.8%) had at least one episode caused by an unidentified trigger.

**Figure 1:**
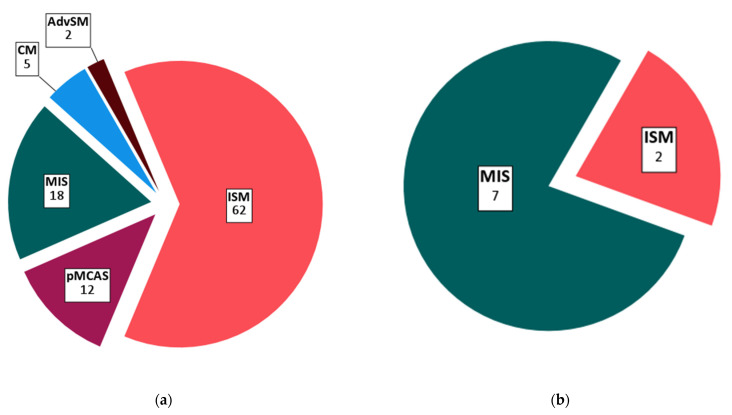
Distribution of primary mast cell disease subtypes: (**a**) Total population (*n* = 99); (**b**) pediatric population (*n* = 9, 12–18 years). Absolute patient counts for each subtype are indicated on the figure. Abbreviations: CM, cutaneous mastocytosis; MIS, mastocytosis in the skin; pMCAS, primary mast cell activation syndrome; ISM, indolent systemic mastocytosis; AdvSM, advanced systemic mastocytosis.

Out of 99 pMCD patients, 5% had a diagnosis of CM, 18% had the intermediate diagnosis of MIS, 12% had pMCAS, 62% had indolent SM, and 2% had advanced SM (Figure 1 and Table 2). We included 9 pediatric pMCD patients aged 12–18 years (7 MIS and 2 ISM). Median baseline tryptase in the pMCD cohort was 18.5 µg/L (range 2–200 µg/L). Most pMCD patients had a history of MCAS (65.7%) and 41.4% had a history of anaphylaxis.

During the study period, 221 vaccine doses were administered in-hospital. Seventy-seven patients (39.3%) received 102 doses of the BNT162b2 vaccine, including 25 patients who received both doses during the study period. In total, 119 patients (53.8%) received the single dose Ad26.COV2-S vaccine.

### 3.2. Occurrence and Risk Factors for Immediate Reactions (IR)

**Figure 2:**
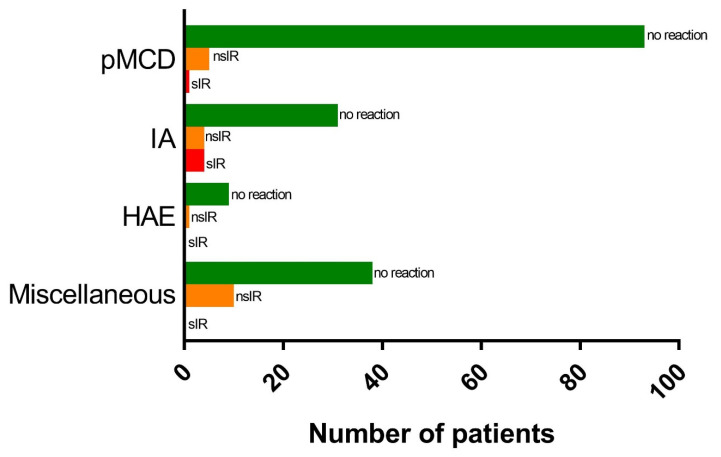
Distribution of immediate reactions among different subgroups. Abbreviations: nsIR, non-severe immediate reaction; sIR, severe immediate reaction; pMCD, primary mast cell disease; IA, idiopathic anaphylaxis; HAE, hereditary angioedema.

Twenty-five IR were observed in a total of 24 patients (12.2%), including 1 patient reacting to both in-hospital BNT162b2 doses (Figure 2 and Table 3). We did not find a significant difference in the incidence of IR between BNT162b2 and Ad26.COV2-S, occurring in 10.8% and 12.4% of doses, respectively (*p* = 0.48). Twenty-three patients reacted to the first in-hospital vaccine dose. One HAE patient developed angioedema of the hand after the second BNT162b2 dose. Three immediate reactors received both doses of BNT162b2 in-hospital. Of these, 2 patients tolerated the second dose without incident and a single IA patient reacted to both doses, reporting apparently unrelated symptoms on both occasions. Interestingly, out of 10 patients with an IR to the first in-hospital dose of BNT162b2, 3 also had a reaction to the first dose of the adenoviral vector vaccine ChAdOx1 (Vaxzevria^®^, Oxford-AstraZeneca) administered prior to the in-hospital vaccination.

Out of the 25 observed IR, 20 (80%) were deemed non-severe (nsIR). nsIR occurred in 10.2% of patients and 9% of administered vaccine doses (10 Ad26.COV2-S and 10 BNT162b2) and consisted of subjective symptoms without observable clinical signs except for one case of ECG-confirmed sinus tachycardia (Table 3). Rescue medication, mainly oral antihistamines, was administered at the discretion of the supervising allergist in 11 cases (55%). All nsIR resolved within 60 min and all patients were discharged within 2 h after vaccination.

Five IR were classified as severe (20%) based on the need for prolonged in-hospital monitoring. Severe IR (sIR) occurred in 2.6% of patients (4 IA and 1 pMCD, all women) and 2.3% of administered vaccine doses (4 Ad26.COV2-S and 1 BNT162b2). Transient arterial hypotension with absence of any other objective clinical signs was observed in 2 IA patients. Rescue medication, including intramuscular adrenalin in one patient, was administered in all sIR cases followed by transfer to the ED for at least 6 h of monitoring. All sIR resolved within 60 min. Blood samples for measurement of acute tryptase levels were obtained in 4/5 sIR patients, including both cases with arterial hypotension. No significant tryptase elevation compared to baseline could be noted in any of the sIR cases and none fulfilled criteria for anaphylaxis.

We performed inferential statistics to look for significant predictors for IR occurrence. IR incidence differed significantly depending on the underlying diagnosis, with patients vaccinated in-hospital for miscellaneous indications (20.8%) and IA patients (17.9%) being at significantly higher risk than HAE patients (10%) and pMCD patients (6.1%) (Chi² (2, *n* = 196) = 8.05, *p* < 0.05). Women were more likely to suffer an IR than men (17.2% vs. 2.9%; *p* < 0.005). As previously mentioned, vaccine type did not influence IR incidence (*p* = 0.48) and median age and prior history of anaphylaxis did not differ significantly between reactors vs. non-reactors (55.7 years vs. 55.0 years; *p* = 0.59 and 45.8% vs. 47.1%; *p* = 0.54, respectively).

No significant differences in IR incidence were observed among the different subgroups of pMCD (*p* = 0.507). Furthermore, compliance with premedication regimens had no statistically significant impact on IR occurrence in our pMCD patients (11.4% in compliant vs. 14.6% in non-compliant patients; *p* = 0.65). pMCD reactors trended towards lower baseline tryptase levels compared to pMCD non-reactors although this trend did not reach statistical significance (15.4 µg/L vs. 23.7 µg/L; *p* = 0.198). Similarly, in pMCD patients we noted a prior history of MCAS in 5/6 reactors (83.3%) vs. 60/93 non-reactors (64.5%) but, possibly due to the low number of reactions observed in this subgroup, this difference was not significant (*p* = 0.457).

### 3.3. Occurrence and Risk Factors for Late Reactions (LR)

Out of 196 patients, 135 (68.9%) responded to the follow-up telephone interview after the first in-hospital dose. A late reaction (LR), defined as any symptom occurring in the first 72 h following vaccination, was reported by 46.6% of responders (Supplementary Material Table S2). There was no significant difference in LR occurrence between patients who received BNT162b2 and those who received Ad26.COV2-S (40.5% vs. 49.5%; *p* = 0.38). Typical vaccine-related side effects were reported by 42.2% of responders, with the most frequent reported symptoms being fatigue (47.3%), myalgia (35.1%), headache (29.8%), and fever (14%). These resolved spontaneously within 72 h in all but 2 cases (3.5%). None of the patients with LR required additional medical assistance.

Seventy-four out of 99 pMCD patients (74.7%) responded to the follow-up interview. Of these, 90.4% reported compliance with the advised antihistamine premedication regimen and 48.6% reported an LR. Thirty pMCD responders (40.5%) reported typical vaccine-related side effects. Interestingly, 10 patients (13.5%) also reported a flare-up of symptoms reminiscent of prior MCAS. All of these patients reported compliance with the advised pre- and post-vaccination medication regimen. The most frequently reported MCAS-like LR were gastro-intestinal (*n* = 3), cardiovascular (*n* = 3), and cutaneous (*n* = 3) symptoms (Supplementary Material Table S3)MCAS-like LR only occurred in patients with a history of such symptoms (10/48 with MCAS history vs. 0/26 without MCAS history; *p* < 0.01). No significant differences in age, sex, baseline tryptase levels, or diagnostic subcategory were noted between pMCD patients with vs. without MCAS-like LR.

## 4. Discussion

We analyzed the occurrence of immediate physician-observed and late patient-reported reactions after COVID-19 vaccination in a population deemed to be at higher risk for IHR including anaphylaxis.

The incidence of IR in our cohort was considered to be high (11.3% of administered doses). This can be explained by the broad definition used for IR, encompassing any symptom. Furthermore, vaccination in a medicalized setting of patients specifically referred due to perceived high risk for IHR most likely induced an expectation bias. Finally, the high IR incidence could be explained in part by the selected underlying conditions. However, several findings argue against the latter. The majority of IR were mild and subjective. No cases of anaphylaxis were identified and for most reactions, including most sIR, alternative diagnoses such as vasovagal syncope, vocal cord dysfunction, and hyperventilation could be made. Furthermore, in all sIR cases, including both cases with arterial hypotension, no transient tryptase elevation was noted, arguing against a mast cell-mediated mechanism.

The reported incidence of anaphylaxis after COVID-19 vaccination varies considerably. In a prospective cohort study of 64,900 healthcare workers receiving mRNA-based vaccines, Blumenthal et al. noted a 2.1% patient-reported incidence of allergic reactions. However, after retrospective review of selected cases by an allergist, the incidence of true Brighton criteria-defined anaphylaxis was determined to be 100-fold lower at 0.025%, equating to 247 cases per million doses [20]. Studies based on passive reporting systems report even lower incidences. Singh et al., using data covering over 230 million doses, calculated an aggregate incidence of 3 cases per million doses for mRNA-based and adenoviral vector vaccines [21]. Similarly, Almuhaid et al. reported an incidence of 5.58 cases per million doses in a pooled analysis of 14 cohorts covering over 26 million doses [22]. This wide range highlights the limitations of both active self-reporting by patients and optional passive reporting by patients and physicians in discriminating between true IHR such as anaphylaxis and nonallergic IR.

High rates of nonallergic reactions from 3.4 to 14% were also reported in several recent cohort studies of physician-observed COVID-19 vaccine-related reactions in healthcare workers. Interestingly, as in our cohort, no anaphylaxis cases were observed [23,24]. In contrast, Shavit et al., reporting on the physician-observed safety of mRNA-based COVID-19 vaccines in an Israeli cohort of 429 allegedly high-risk patients, defined as a history of drug or vaccine-induced anaphylaxis or history of allergy to multiple triggers, reported an anaphylaxis incidence of 0.3% (1/429). The authors did not use a standardized anaphylaxis definition and did not perform serial serum tryptase measurements which could explain the differences with our findings. Nevertheless, in line with our work, they also observed a high frequency of non-allergic reactions (2.4%) with a female predominance [25]. Interestingly, the highest risk of IR in our study was also observed in patients with miscellaneous indications for in-hospital vaccination, including mostly patients with a history of often poorly specified reactions to vaccines, drugs, or undetermined triggers.

Our population included the largest pMCD vaccination cohort reported to date, including nine children. We observed no anaphylaxis in this group, highlighting the safety of both mRNA-based and adenoviral vector vaccines in pMCD patients. Similarly, two smaller studies reporting on mRNA-based COVID-19 vaccine safety in a total of 20 pMCD patients with both cutaneous and systemic forms reported no IHR or anaphylaxis [26,27]. Interestingly, we did note the occurrence of late MCAS-like symptoms in 13.5% of pMCD patients, all of which occurred in patients with a history of such symptoms. All were mild and non-anaphylactic in nature and probably allude to a non-specific vaccine-induced event, as has been described with other non-specific triggers [28]. A 2017 cohort study by Zanoni et al. retrospectively evaluated the safety and tolerability of different vaccines in 72 pMCD patients including 30 children. They observed no anaphylaxis but did note flare-up of cutaneous MCAS in four children [29]. In a recent statement by the U.S. and European pMCD societies, the potential risk of IHR following COVID-19 vaccination in pMCD patients was acknowledged and the use of antihistamine premedication was advised [11]. While not powered to assess the effect of premedication, our findings indirectly support the rationale for antihistamine use during the peri-vaccination period.

In accordance with guidance issued by the US CDC, European Medicines Agency (EMA), and UK Medicines and Healthcare products Regulatory Agency (MHRA), the European Academy of Allergy and Clinical Immunology (EAACI) stated in a position paper that formal contraindications for COVID-19 vaccination should be limited to allergy to a vaccine component or allergic reactions to the first vaccine dose. The authors suggest that these patients should always be referred to an allergist for evaluation prior to vaccination [8,9,10,30]. Whereas national guidelines are generally in agreement concerning these contraindications, the identification of other groups at risk for vaccine IHR and the precautions to be taken in those cases remain a point of contention. The EAACI paper advises in-hospital vaccination in patients with uncontrolled asthma or mast cell disease whereas the Belgian guidelines expanded this to patients with idiopathic anaphylaxis and hereditary angioedema but did not include patients with uncontrolled asthma [7,30]. The French Allergy Society (SAR) recommendations state that precautions in mast cell disease, idiopathic anaphylaxis, and hereditary angioedema should be individualized [8,30]. Our analysis indicates that in these three groups, COVID-19 vaccination appears to be safe and that, in most patients, vaccination can be performed in a routine setting with a 30 min observation period, provided that no other formal contraindications are present. In accordance with previously issued guidelines, resuscitation equipment should be readily available, and personnel should be trained in the recognition and management of IHR [8,9,10,30]. For mast cell disease patients, in the absence of randomized prospective evidence, use of antihistamine premedication remains prudent [11]. Nevertheless, if these conditions cannot be met or if the patient is at risk of deferral from vaccination in a routine setting, in-hospital vaccination can be considered as a safe and valid alternative.

The retrospective design of our study could be considered a disadvantage since differences in clinical management and the decision to administer rescue medication when reactions were observed might have influenced outcome. Nevertheless, vaccination occurred in a standardized setting and assessment of reactions occurred in real-time by physicians with expertise in diagnosis and management of IHR and using validated criteria. The retrospective approach was also advantageous in the sense that it limited additional expectation bias in patients. While the small sample size means our study may be underpowered to detect rare events, we demonstrate that, even in populations deemed at high risk, incidence of anaphylaxis appears to be low. The observed reactions were mostly subjective and we hypothesize that this was, at least in part, due to a nocebo effect induced by vaccination in a medicalized setting.

## 5. Conclusions

Vaccination with both mRNA-based and adenoviral vector COVID-19 vaccines was safe and well-tolerated in patients with pMCD, IA, and HAE. Female patients and those with IA and miscellaneous indications had the highest likelihood of experiencing an IR. No cases of anaphylaxis occurred. The relatively high incidence of mild nonallergic and/or subjective reactions observed during in-hospital vaccination suggests a nocebo effect.

For pMCD patients, in the absence of placebo-controlled trials, use of antihistamine premedication remains prudent, and patients should be informed of the potential for MCAS flare-ups post-vaccination and instructed in self-management strategies.

The nocebo effect and additional strain on medical resources is a drawback of monitored in-hospital vaccination of specific populations. However, in a subset of patients, in-hospital vaccination provided the means for safe vaccination without which some patients might not have become vaccinated.

## Figures and Tables

**Table 1:** Baseline population characteristics.

Characteristic No. (%)						
Diagnostic Category		Total *n* = 196	pMCD *n* = 99/196 (50.5%)	IA *n* = 39/196 (19.9%)	HAE *n* = 10/196 (5.1%)	Miscellaneous *n* = 48/196 (24.5%)
Age Median (range), y		51.5 (12–84)	48 (12–82)	58 (27–84)	40.5 (20–68)	52.5 (21–73)
Baseline tryptase ^†^ Median (range), µg/L		8.5 (1.5–200)	18.5 (2–200)	4.95 (1.8–20.6)	4.85 (3.5–7.6)	4.8 (1.5–20.9)
Sex	Male	68 (34.7)	42 (42.4)	9 (23.1)	6 (60)	11 (22.9)
	Female	128 (65.3)	57 (57.6)	30 (76.9)	4 (40)	37 (77.1)
Anaphylaxis history	All causes	104 (53.1)	41 (41.4)	39 (100)	0 (0)	24 (50)
	Unknown trigger	57 (29.1)	13 (13.1)	39 (100)	0 (0)	5 (10.4)
	Venom	27 (13.7)	23 (23.2)	2 (5.1)	0 (0)	2 (4.2)
	PEG	11 (5.6)	0 (0)	0 (0)	0 (0)	11 (22.9)
Vaccine used	Ad26.COV2-S	119 (60.7)	58 (58.6)	29 (74.4)	4 (40)	28 (58.3)
	BNT162b2	77 (39.3)	41 (41.4)	10 (25.6)	6 (60)	20 (41.7)

^†^ upper reporting limit for serum tryptase assay is 200 µg/L. Abbreviations: pMCD, primary mast cell disease; IA, idiopathic anaphylaxis; HAE, hereditary angioedema; PEG, polyethylene glycol.

**Table 2:** Baseline characteristics of primary mast cell disease patients.

Characteristic No. (%)							
pMCD Subcategory		Total *n* = 99	CM *n* = 5/99 (5%)	MIS *n* = 18/99 (18%)	pMCAS *n* = 12/99 (12%)	ISM *n* = 62/99 (62%)	AdvSM *n* = 2/99 (2%)
Age Median (range), y		48 (12–82)	38 (22–63)	20.5 (12–62)	50.3 (33–72)	53.2 (14–82)	64.5 (63–66)
Baseline tryptase ^†^ Median (range), µg/L		18.5 (2–200)	9.6 (4.9–16.3)	6.2 (2.3–21.9) ^‡^	7.7 (2–21.4)	29.6 (3–200)	200 (200–200)
Sex	Male	42 (42.4)	1 (20)	9 (50)	6 (50)	25 (40.3)	1 (50)
	Female	57 (57.6)	4 (80)	9 (50)	6 (50)	37 (59.7)	1 (50)
c-KIT D816V mutation present		68/81 (84) ^‡^	2 (40)	2/3 (66.7) ^‡^	6 (50)	57/60 (95) ^‡^	1/1 (100) ^‡^
Anaphylaxis history		41 (41.4)	1 (20)	0 (0)	8 (66.7)	31 (50)	1 (50)
MCAS history		65 (65.7)	4 (80)	10 (55.6)	8 (66.7)	41 (66.1)	2 (100)
Vaccine used	Ad26.COV2-S	58 (58.6)	2 (40)	2 (11.1)	8 (66.7)	44 (71)	2 (100)
	BNT162b2	41 (41.4)	3 (60)	16 (88.9)	8 (33.3)	44 (29)	0 (0)

^†^ upper reporting limit for serum tryptase assay is 200 µg/L; ^‡^ calculated for all informative cases; Abbreviations: pMCD, primary mast cell disease; CM, cutaneous mastocytosis; MIS, mastocytosis in the skin; pMCAS, primary mast cell activation syndrome; ISM, indolent systemic mastocytosis; AdvSM, advanced systemic mastocytosis; MCAS, mast cell activation symptoms.

**Table 3:** Immediate reactions.

	Patient Type	Sex, Age (y)	Anaphylaxis History	Other History	Vaccine	Dose	Symptoms	Rescue Medication	Duration	Tryptase Elevation
**sIR** *n* = 5	pMCD (ISM)	F, 53	Yes (venom, shrimp)	-	BNT162b2	First dose	Tachycardia	H1	<10 min	-
	IA	F, 52	Yes (unknown)	CSU	Ad26.COV2-S	Only dose	Syncope with art. hypotension	H1, IM adrenalin, IV fluids	<20 min	No
	IA	F, 58	Yes (unknown)	AD, ARC asthma	Ad26.COV2-S	Only dose	Shortness of breath, hoarseness, cough (probable VCD)	H1, IV CS, ICS, SABA	<60 min	No
	IA	F, 73	Yes (unknown)		Ad26.COV2-S	Only dose	Syncope with art. hypotension	IV fluids	<10 min	No
	IA	F, 48	Yes (unknown)	Crohn’s disease, CSU	Ad26.COV2-S	Only dose	Generalized pruritus, nausea	H1, IV CS	<60 min	No
**nsIR** *n* = 20	pMCD (MIS)	F, 12	No	-	BNT162b2	First dose ^†^	Lightheadedness, abdominal pain, pharyngeal discomfort	None	<60 min	-
	pMCD (pMCAS)	F, 53	Yes (unknown)	Asthma, ARC,	Ad26.COV2-S	Only dose	Metallic taste	None	<60 min	-
	pMCD (pMCAS)	M, 44	Yes (venom, unknown)	-	Ad26.COV2-S	Only dose	Chest discomfort	None	<20 min	-
	pMCD (ISM)	F, 65	No	-	Ad26.COV2-S	Only dose	Tinnitus, auricular pressure sensation	None	<30 min	-
	pMCD (ISM)	F, 77	No	-	Ad26.COV2-S	Only dose	Sinus tachycardia	H1	<60 min	-
	IA ^+^	F, 41	Yes (unknown)	-	BNT162b2	First dose ^§^	Shortness of breath, nausea	SABA	<60 min	-
						Second dose ^§^	Diffuse pruritus	H1	<60 min	-
	IA	M, 44	Yes (unknown)	ARC	Ad26.COV2-S	Only dose	Vasovagal syncope	None	<20 min	-
	IA	F, 65	Yes (venom, unknown)	-	Ad26.COV2-S	Only dose	Headache, blurred vision	None	<30 min	-
	HAE	F, 44	No	VCD	BNT162b2	Second dose	Angioedema hand	C1-inhibitor, icatibant	<60 min	-
	Miscellaneous	F, 27	No	VCD, asthma	BNT162b2	First dose ^‡^	VCD	IV CS, SABA, inhaled adrenalin	<60 min	-
	Miscellaneous	F, 32	No	-	BNT162b2	First dose ^‡^	Shortness of breath, glowing sensation, lightheadedness	H1	<60 min	-
	Miscellaneous	F, 43	No	-	BNT162b2	First dose ^‡^	Lightheadedness	None	<15 min	-
	Miscellaneous	F, 37	No	-	BNT162b2	First dose	Shortness of breath	SABA	<60 min	-
	Miscellaneous	F, 62	Yes (influenza vaccine)	-	BNT162b2	First dose	Pharyngeal pruritus	H1	<15 min	-
	Miscellaneous	F, 47	Yes (venom, influenza vaccine)	-	BNT162b2	First dose	Generalized pruritus, headache	H1	<60 min	-
	Miscellaneous	F, 60	No	-	Ad26.COV2-S	Only dose	Hyperventilation, anxiety	None	<20 min	-
	Miscellaneous	F, 72	No	Atrial fibrillation	Ad26.COV2-S	Only dose	Headache	None	<20 min	-
	Miscellaneous	F, 76	No	COPD	Ad26.COV2-S	Only dose	Shortness of breath, headache	SABA	<30 min	-
	Miscellaneous	F, 51	Yes (BNT162b2)	AD, asthma	Ad26.COV2-S	Only dose	Shortness of breath, chest discomfort	H1	<60 min	-

^†^ first BNT162b2 dose was administered in-hospital after previous reaction to first ChAdOx1 dose in vaccination center; ^‡^ second dose was also administered in-hospital and tolerated; ^§^ the same patient reacted to both doses administered in-hospital. Abbreviations: sIR, severe immediate reaction; nsIR, non-severe immediate reaction; pMCD, primary mast cell disease; ISM, indolent systemic mastocytosis; MIS, mastocytosis in the skin; IA, idiopathic anaphylaxis; F, female; M, male; CSU, chronic spontaneous urticaria; AD, atopic dermatitis; ARC, allergic rhinoconjunctivitis; VCD, vocal cord dysfunction; COPD, chronic obstructive pulmonary disease; H1, H1 antihistamine; IM, intramuscular; IV, intravenous; CS, corticosteroids; ICS, inhaled corticosteroids; SABA, inhaled short-acting beta-2-agonist.

## Data Availability

The data presented in this study are available on request from the corresponding author. The data are not publicly available due to privacy and ethical restrictions.

## Supplementary Materials

The following supporting information can be downloaded at: https://www.mdpi.com/article/10.3390/vaccines10020286/s1, Table S1: Overview of population invited for in-hospital vaccination for miscellaneous indications; Table S2: Overview of late reactions; Table S3: Overview of MCAS-like reactions; protocol for management of immediate reactions.
