# Supplementary material for: COVID-19 Vaccination Safety and Tolerability in Patients Allegedly at High Risk for Immediate Hypersensitivity Reactions
Source: Vaccines (Basel). 2022 Feb 14;10(2):286. doi: 10.3390/vaccines10020286 (PMC8879861; doi:10.3390/vaccines10020286)
Supplement: Supplementary file 1 [file vaccines-10-00286-s001.zip › vaccines-1544412-supplementary.pdf]

Supplementary Materials

Table S1 – Overview of population invited for in-hospital vaccination for miscellaneous indications

Table S2 – Overview of late reactions

Table S3 – Overview of MCAS-like late reactions

Protocol for management of immediate reactions

References

Table S1 – Overview of population invited for in-hospital vaccination for miscellaneous indications

Subgroup	Sex	Age (y)	Triggers for previous reactions	Administered vaccine	IR	Baseline tryptase (kU/L)
**History of reactions to non-COVID-19 vaccines** n = 12	F	62	influenza vaccine[+]	BNT162b2	nsIR	6,2
	F	49	influenza vaccine[+]	BNT162b2	-	-
	F	72	influenza vaccine	Ad26.COV2-S	nsIR	-
	F	47	influenza vaccine[+], wasp venom	BNT162b2	nsIR	2,4
	F	64	influenza vaccine (Pandemrix)[+]	BNT162b2	-	-
	F	67	influenza vaccine (Pandemrix), pneumococcal vaccine (Prevenar)[+]	BNT162b2	-	-
	F	42	influenza vaccine, multiple other drugs	BNT162b2	-	-
	F	59	pneumococcal vaccine (Pneumovax)	Ad26.COV2-S	-	1,5
	F	63	multiple vaccines, multiple drugs	Ad26.COV2-S	-	3,1
	F	64	multiple vaccines, multiple drugs[+]	Ad26.COV2-S	-	-
	M	34	tetanus vaccine, meningococcal vaccine[+]	BNT162b2	-	-
	M	42	unknown vaccine	Ad26.COV2-S	-	7,3
**Skin test confirmed PEG allergy** n = 11	F	55	ibuprofen (PEG400) [#]	Ad26.COV2-S	-	3,1
	F	51	BNT162b2 (PEG2200)	Ad26.COV2-S	nsIR	5,7
	F	21	Depomedrol (PEG3350) [#]	Ad26.COV2-S	-	3,3
	F	69	Depomedrol (PEG3350) [#]	Ad26.COV2-S	-	8,2
	F	60	Depomedrol, Moviprep (PEG3350) [#]	Ad26.COV2-S	-	-
	F	60	Kleanprep (PEG3350) [#]	Ad26.COV2-S	-	-
	F	73	Forlax (PEG4000) [#]	Ad26.COV2-S	-	8,5
	M	53	Depomedrol (PEG3350) [#]	Ad26.COV2-S	-	4,4
	M	39	Depomedrol (PEG3350) [#]	Ad26.COV2-S	-	6,3
	M	56	Diprophos (PEG3350) [#]	Ad26.COV2-S	-	4,8
	M	67	Proflox (PEG4000) [#]	Ad26.COV2-S	-	4,2
	F	27	ChAdOx2	BNT162b2	nsIR	1,7

**History of reactions to first COVID-19 vaccine dose** n = 9	F	32	ChAdOx2+	BNT162b2	nsIR	4,7
	F	43	ChAdOx2+	BNT162b2	nsIR	-
	F	51	ChAdOx2+	BNT162b2	-	5,8
	F	48	ChAdOx2+	BNT162b2	-	6,1
	F	73	BNT162b2+	BNT162b2	-	-
	F	40	BNT162b2+	BNT162b2	-	-
	F	44	BNT162b2+	BNT162b2	-	-
	F	65	BNT162b2+	BNT162b2	-	5,9
**Other** n = 16	F	58	PEG-containing drug (Aethoxysklerol)+	Ad26.COV2-S	-	5,9
	F	50	PEG-containing drug (Depo-Medrol)+	Ad26.COV2-S	-	4,7
	F	52	PEG-containing drug (Diprophos)+	Ad26.COV2-S	-	-
	F	60	multiple drugs+	Ad26.COV2-S	nsIR	3,6
	F	73	multiple drugs	Ad26.COV2-S	-	3,6
	F	65	multiple drugs+	Ad26.COV2-S	-	4,5
	F	76	multiple drugs	Ad26.COV2-S	nsIR	-
	F	26	anaphylaxis-like reaction to single unknown trigger	BNT162b2	-	3,0
	F	50	anaphylaxis-like reaction to single unknown trigger	Ad26.COV2-S	-	4,8
	F	37	possible hereditary angioedema (evaluation ongoing at time of vaccination)	BNT162b2	nsIR	-
	F	22	complex primary food allergy	BNT162b2	-	5,6
	M	48	PS80-containing drug (unspecified)+	BNT162b2	-	-
	M	71	PEG-containing drug (Depo-Medrol)+	Ad26.COV2-S	-	7,2
	M	47	anaphylaxis to PEG-containing drug (Diprophos) §	Ad26.COV2-S	-	-
	M	49	anaphylaxis-like reaction to single unknown trigger	Ad26.COV2-S	-	4,8
	M	69	anaphylaxis-like reaction to single unknown trigger	Ad26.COV2-S	-	20,9*

Abbreviations: IR, immediate reaction; F, female; M, male; PEG, polyethylene glycol; PS80, polysorbate 80; nsIR, non-severe immediate reaction

+ negative skin tests for PEG and PS80
§ index reaction was anaphylaxis after Diprophos IM; negative skin testing for PEG and PS80 (excipients), positive for bethamethasone dipropionate (active ingredient)
# previously described in Ieven *et al.*[1]
* c-KIT D816V mutation was negative

Table S2 – Overview of late reactions

Characteristic		No. (%)				
**Diagnostic category**		**Total**	**pMCD**	**IA**	**HAE**	**Miscellaneous**
**Follow-up response**		135/196 (68.9)	76/99 (76.8)	30/39 (79.9)	7/10 (70)	22/48 (45.8)
**Late reaction**						
	**Overall**	57/135 (42.2)	36/76 (47.4)	13/30 (43.3)	2/7 (28.6)	12/22 (54.5)
	**Vaccine-related**	-	30/76 (39.5)	-	-	-
	**MCAS-like**	-	10/76 (13.2)	-	-	-

Abbreviations: pMCD, primary mast cell disease; IA, idiopathic anaphylaxis; HAE, hereditary angioedema; MCAS, mast cell activation symptoms

Table S3 - Overview of MCAS-like late reactions

Diagnosis	Sex	Age	Baseline tryptase (kU/L)	Vaccine	MCAS-like LR symptoms	Other LR symptoms	Prior MCAS symptoms	Onset	Duration	Management
ISM	F	63	45	Ad26.COV2-S	cutaneous		cutaneous	> 24 hours	< 72 hours	H1, LTRA
ISM	F	56	48,3	Ad26.COV2-S	gastro-intestinal		gastro-intestinal, cardiovascular	> 12 hours	< 72 hours	H1
ISM	F	65	71,9	Ad26.COV2-S	gastro-intestinal		cutaneous, gastro-intestinal	> 24 hours	< 72 hours	oral cromolyn
ISM	F	50	70	Ad26.COV2-S	cutaneous		cutaneous, constitutional	< 6 hours	< 6 hours	-
ISM	F	57	168	Ad26.COV2-S	cutaneous		cutaneous, cardiovascular, gastro-intestinal	> 24 hours	< 72 hours	-
ASM	F	63	> 200	Ad26.COV2-S	constitutional		constitutional, cardiovascular	< 6 hours	< 24 hours	H1
pMCAS	M	53	21,4	Ad26.COV2-S	cardiovascular	Fatigue, myalgia	cardiovascular, gastro-intestinal	> 6 hours	< 24 hours	-
ISM	M	36	19,4	BNT162b2	cardiovascular	fatigue, headache	cardiovascular	> 6 hours	< 24 hours	-
ISM	F	54	7,3	Ad26.COV2-S	cardiovascular	fatigue, headache, fever	cardiovascular	< 6 hours	< 48 hours	-
CM	F	35	14,7	BNT162b2	gastro-intestinal		cutaneous, gastro-intestinal	> 12 hours	< 24 hours	-

Abbreviations: MCAS, mast cell activation symptoms; ISM, indolent systemic mastocytosis; ASM, agressive systemic mastocytosis; pMCAS, primary mast cell activation syndrome; CM, cutaneous mastocytosis; H1, H1-anthistamine

Gastro-intestinal MCAS: diarrhoea, abdominal cramping, dyspepsia
Cardiovascular MCAS: flushing, palpitations, dizziness/presyncope
Cutaneous MCAS: increased whealing, erythema and pruritus of existing lesions
Constitutional MCAS: general malaise, fever, myalgia

Protocol for management of immediate reactions

The protocol for the management of immediate vaccine reactions was outlined in a preapproved internal standard operating procedure (SOP) document generated prior to initiation of the in-hospital vaccination of alleged high-allergy risk patients.

In brief:

- All nursing staff were instructed in the recognition of early mild and major signs of anaphylaxis as well as the most important differential diagnoses (including vagal syncope and hyperventilation), prior to initiation of the vaccination protocol.
- Upon patient admission to the vaccination unit, baseline vital signs were checked and registered and IV access was obtained.
- Nursing staff were present in close proximity to the patients during a mandatory 30-minute post-vaccination observation window. If a patient reported any symptoms, nurses were instructed to immediately inform the supervising allergist(s) and proceed to check patient blood pressure and oxygen saturation.
- The supervising allergist(s) would perform a clinical evaluation of the patient.
- Based on these findings, the evaluating allergist(s) would decide whether to administer rescue medication and whether to transfer the patient to a separate on-site observation room with facilities for supplemental oxygen administration and continuous heart rhythm monitoring.
- In case of a severe reaction with respiratory and/or hemodynamic compromise, basic life support would be initiated and a code would be called to summon the internal medical response unit from the emergency department to performed advanced life support if required.
- Stable patients who were deemed to require prolonged monitoring (i.e. those with potentially severe initial symptoms, deemed to be at particular risk for a more severe reaction or with slow and/or incomplete resolution of symptoms after 1-2 hours of on-site observation) were transferred to the emergency department for prolonged monitoring (6 hours).

References

1. Ieven T, Van Weyenbergh T, Vandebotermet M, et al. Tolerability of polysorbate 80-containing COVID-19 vaccines in confirmed polyethylene glycol-allergic patients. *J Allergy Clin Immunol Pract*. 2021 Oct 6;S2213-2198(21)01106-5. doi: 10.1016/j.jaip.2021.09.039.
